# Thermal Characterization of Phantoms Used for Quality Assurance of Deep Hyperthermia Systems

**DOI:** 10.3390/s20164549

**Published:** 2020-08-13

**Authors:** Laura Farina, Kemal Sumser, Gerard van Rhoon, Sergio Curto

**Affiliations:** 1 Translational Medical Device Lab, National University of Ireland Galway, H91 TK33 Galway, Ireland; laura.farina@nuigalway.ie; 2 CURAM, SFI Research Centre for Medical Devices, National University of Ireland Galway, H91 TK33 Galway, Ireland; 3 Erasmus MC Cancer Institute, Department of Radiation Oncology, University Medical Center Rotterdam, 3015 GD Rotterdam, The Netherlands; k.sumser@erasmusmc.nl (K.S.); g.c.vanrhoon@erasmusmc.nl (G.v.R.)

**Keywords:** thermal properties, hyperthermia, deep hyperthermia, QA phantoms, thermal properties analyzer device, thermal properties sensitivity evaluation

## Abstract

Tissue mimicking phantoms are frequently used in hyperthermia applications for device and protocol optimization. Unfortunately, a commonly experienced limitation is that their precise thermal properties are not available. Therefore, in this study, the thermal properties of three currently used QA phantoms for deep hyperthermia are measured with an “off-shelf” commercial thermal property analyzer. We have measured averaged values of thermal conductivity (*k* = 0.59 ± 0.07 Wm^−1^K^−1^), volumetric heat capacity (*C =* 3.85 ± 0.45 MJm^−3^K^−1^) and thermal diffusivity (*D =* 0.16 ± 0.02 mm^2^s^−1^). These values are comparable with reported values of internal organs, such as liver, kidney and muscle. In addition, a sensitivity study of the performance of the commercial sensor is conducted. To ensure correct thermal measurements, the sample under test should entirely cover the length of the sensor, and a minimum of 4 mm of material parallel to the sensor in all directions should be guaranteed.

## 1. Introduction

Tissue mimicking models (phantoms) have been proposed and are increasingly being adopted in the design, optimization and quality assurance of medical devices to guarantee their correct performance in clinical applications. In particular, multi-modal phantoms have been developed and are used in electromagnetic-based hyperthermia applications, such as deep or superficial hyperthermia and thermal ablation [1,2,3,4,5,6,7,8]. Such phantoms are required to mimic the target portion of human body of interest from an electromagnetic perspective, but also from a thermal point of view. While the dielectric properties of such phantoms have been extensively evaluated, the thermal properties of the same are in general not available. In this study, we report the results of our work to thermally characterize three different types of phantoms that are frequently used in quality assurance (QA) of deep hyperthermia systems. 

Current popular tissue-mimicking models in use for QA of deep hyperthermia systems are the high-viscosity phantom presented in [5], the semi-solid phantom presented in [6] and the solid phantom presented in [7]. The high-viscosity phantom based on premixed wallpaper paste is proposed to perform a quality assurance evaluation of the BSD2000-3D MR-compatible hyperthermia applicator. The phantom is shown to be an excellent tool to evaluate the steering accuracy of the hyperthermia device evaluated with MR imaging [5]. The semi-solid phantom is based on wallpaper paste powder [6]. This phantom has been successfully used in the evaluation of deep hyperthermia applicators and recently on a 70 MHz waveguide applicator for the treatment of superficial tumors with deep infiltration [8,9]. More recently, a solid phantom based on agar and sodium benzoate has been proposed to perform a multi-institutional evaluation of MR Thermometry accuracy for deep-pelvic MR Hyperthermia systems. This phantom has a human representative shape and contains bone structures. It is considered a valuable tool for performing QA evaluation as well as for training the hyperthermia clinical team [7].

Since the aim of hyperthermia application is to obtain a controlled temperature increase in the target tissue or area, such phantoms should besides having the correct electrical characteristics, also possess tissue representative thermal properties. The investigation of the thermal properties of the body areas of interest has mainly focused so far on tissue perfusion [10,11]. However, thermal properties, such as thermal conductivity, thermal diffusivity and volumetric heat capacity also contribute to the thermal process occurring in the tissue during exposure to an electromagnetic field, and they too define the final temperature increase and the extension of the heat distribution. These properties have been less accurately investigated, also due the lack of good reference values. Thermal properties of biological tissues have been investigated in past decades to better understand and characterize their influence and their role in the hyperthermia treatments [12]. Unfortunately, the different methodologies adopted and the different study designs have resulted in unsatisfying and incoherent results [13].

For this reason, recently, the European electromagnetic hyperthermia community (https://www.cost.eu/actions/CA17115/#tabs|Name:overview) and the American Society of Mechanical Engineers (ASME) have investigated the guidelines for such measurements. In past years, different authors have successfully proposed the use of the transient hot-wire technique reporting consistent results. In [14,15,16], the commercial analyser from METER Group, Inc. USA (Pullman, WA, USA), implementing the same transient hot-wire technique, was used to assess the thermal properties of ex vivo liver tissue as a function of the temperature increase up to ablative temperatures. In [17], the same methodology has been used to assess the changes in thermal properties of ex vivo kidney tissue at ablative temperatures. In [18], the same device has been adopted to characterize the thermal properties of different ex vivo biological tissues (liver, kidney, muscle and lung) at room and body temperature.

One important and well-known limitation of this commercial device is the dimensions of the sensor and thus the dimensions required for the biological sample to accurately perform the measurements. In the system’s manual, it is recommended to “allow a minimum of 15 mm of material parallel to the sensor in all directions to avoid errors.” For the sensor to be able to measure thermal conductivity, volumetric heat capacity and thermal diffusivity (the dual needle-sensor [16]), the minimum dimensions required for the sample under test (about 25–40 cm^3^) need to be bigger than the available biological sample. Some biological samples of interest of the electromagnetic hyperthermia community, such as small glands or tissue obtained from pathology, can be substantially smaller than the recommended dimensions.

In this study, we used the same commercial analyzer to measure the thermal properties of the three phantoms earlier discussed (high-viscosity, semi-solid and solid) and employed in the assessment of deep hyperthermia systems. Additionally, we have measured dielectric and MRI properties of these phantoms. Moreover, we used one of these phantoms, the solid one, to conduct a sensitivity study of the sensor to assess its limit of usability in terms of dimensions of the sample.

## 2. Materials and Methods

### 2.1. Phantoms Preparation and Assessment (Dielectric Properties)

Three different phantoms have been evaluated. The high-viscosity phantom has been prepared using premixed wallpaper paste with deionized water as described in Mulder et al. [5]. The semi-solid phantom has been prepared using powder wallpaper paste, salt and deionized water as described by Schneider et al. [6]. The solid phantom has been prepared using agar, sodium benzoate and deionized water as described in Curto et al. [7]. Details regarding the exact weight percentage of the phantom materials are mentioned in the respective references [5,6,7].

The DAK dielectric assessment kit (Speag, Zurich, Switzerland) was used to measure the dielectric properties of the different phantoms in the frequency range from 50 MHz to 500 MHz. The DAK-12 probe was connected to a Rohde and Schwarz ZNC 3 vector network analyzer (Rohde & Schwarz, Munich, Germany) with a low-loss coaxial cable. A holder for the dielectric probe and a lift for the movement of the material under test completed the set up. Before each set of measurements, the dielectric measurement device was calibrated using three different loads: open circuit, short circuit and deionized water. Measurements were performed in each phantom in triplicate at room temperature. 

### 2.2. MR Relaxometry Measurement Protocols

Longitudinal (T1) and transversal (T2) MR relaxation times of the three phantoms have been evaluated at 1.5 T using a 450w MR scanner (GE Healthcare, Waukesha, WI, USA). T1 relaxation times were measured with an inversion recovery (IR) turbo spin echo sequence with the following sequence parameters: repetition time (TR) = 3000 ms, inversion time (TI) = (50, 100, 150, 200, 250, 500, 750, 1000, 1250, 1500, 1750, 2000, 2250, 2500) ms, echo time (TE) = 13 ms, field of view (FOV) = 360 mm, slice thickness = 4 mm, acquisition matrix 256 × 256, number of excitations (NEX) = 1 [19]. The T1 relaxometry data were analyzed using MATLAB (R2018b, The MathWorks Inc., Natick, USA) script by Barral et al. [20] based on [21]. T2 relaxation times were measured with a spin echo sequence with the following sequence parameters: TR = 2000 ms, TE = (9, 15, 25, 35, 55, 75, 95, 115, 135, 155, 195) ms, FOV = 360 mm, slice thickness = 4 mm, acquisition matrix 128 x 128, NEX = 1 [19]. The T2 relaxation rates were calculated by fitting a mono-exponential signal decay model using the nonlinear curve fitting function lsqcurvefit of Matlab with an in-house developed script. Relaxation measurements were repeated once. The measured values were calculated per voxel and reported as mean and standard deviation over ~1600 voxels. 

### 2.3. Thermal Measurements Set Up and Protocols

A commercial thermal properties analyzer device (TEMPOS, Meter Group, Inc., Pullman, WA, USA, accuracy: 10%) was used in this study to investigate the thermal properties of the phantoms. The device is equipped with different sensors: the single-needle sensor (KS-3) is designed to measure thermal conductivity in liquid and semi-solid materials, while the dual-needle sensor (SH-3) is designed to measure the thermal properties (thermal conductivity, thermal diffusivity and volumetric heat capacity) in solid materials. The KS-3 is made of one needle 60 mm long and 1.3 mm in diameter. The needle is heated by the device for 30 s, and then it measures the temperature increase obtained in the material under test to derive its thermal conductivity. The SH-3 sensor is made of two needles, 30 mm long and 1.3 mm in diameter, 6 mm spaced. The dual-needle sensor mechanism consists in a heating period followed by a cooling period. Heat is applied for 30 s to one of the needles of the sensor (heating needle), while the other needle (monitoring needle) records the changes in temperature due to heat transfer from the heating needle to the material under test. The TEMPOS device uses an algorithm to determine the thermal conductivity and thermal diffusivity of the material under test by a least square procedure; then, volumetric heat capacity is derived [16]. The dual-needle sensor is not suitable for measurements in liquid materials due to the convective phenomena induced by the measuring process in between the two needles, which alter the accuracy of the results.

The single-needle sensor was used to conduct thermal conductivity measurements of all three phantoms; additionally, the dual-needle sensor was adopted to measure the thermal properties of the solid phantom. The sensors were held in position within the sample for approximately 15 min before taking the first measurement; afterwards, measurements were conducted with a time interval of at least 10 min, in order to reach equilibrium temperature. At the beginning and at the end of the measurement sessions, the sensors performances were validated using the dedicated tools supplied by the manufacturer. The thermal properties of a glycerin sample, for the single-needle sensor, and of a dieldrin block, for the dual-needle sensor, were measured, and their compliance with the reference values was verified.

**Figure 1:**
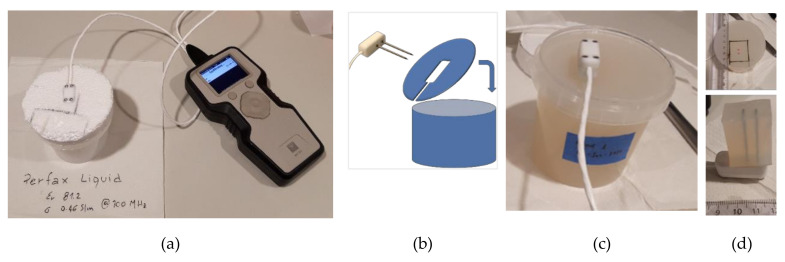
Measurement set up. (**a**) Photography showing that the thermal property analyzer is connected to the sensor; the sensor is immersed in the high-viscosity phantom and held in place by a dedicated fixture. (**b**) Sketch of the fixture showing the opening where the sensor is placed. (**c**) Without fixture. (**d**) Top: the dimension of the sample where decreased keeping fixed the sensor position; bottom: the S4 sample, i.e., the sample with 8 mm of material parallel to the sensor in all directions.

Measurements were conducted on each phantom over 10 days to investigate the possible changes occurring in the mixtures with time: fast changes were observed for the first four days and then, in the second week, stabilization of the values was assessed. The phantoms were stored in sealed containers; the lid of each container was replaced with a dedicated home-made fixture able to held in place the sensors during the measurements, as shown in Figure 1. Three to five measurements were conducted on each phantom per day. 

Then, the solid phantom was used to conduct a sensitivity study with the dual-needle sensor. Given the manufacturer recommendation, the minimum dimension required for the sample under test is 30 × 36 × 30 mm. Measurements were first performed in a bulky sample that satisfied the requirements. Then, the dimensions of material parallel to the sensor in all directions were decreased step by step from 15 mm down to 2.5 mm, as detailed in Table 1 and shown in Figure 1d.

## 3. Results and Discussion

The phantoms were assessed from a dielectric point of view: the phantoms’ dielectric properties matched the expected ones at the deep hyperthermia frequencies (70 and 100 MHz), as detailed in Table 2. The values measured at the frequency of 434 MHz, which is an industry, science and medicine (ISM) frequency in Europe and commonly used for superficial hyperthermia treatments, are reported too.

### 3.1. MR Relaxometry Properties of the Phantoms

The relaxation times of the phantoms are given in Table 3. All three phantoms have T1 relaxation times comparable to the water T1 relaxation time. Hence, T1 relaxations times of the phantoms are longer than the T1 relaxation times of muscle (1130 ms at 1.5 T) [22]. This result was expected since these phantoms are water based and do not include any T1 shortening agent such as copper sulfate or nickel oxide. In a similar fashion, high-viscosity and semi-solid phantoms have T2 relaxation times comparable to the water T2 relaxation time. However, agar is a known T2 shortening agent [23], therefore the solid phantom has a shorter T2 relaxation time compared to high-viscosity and semi-solid phantoms (67 ms vs 870 and 742 ms). Still, even the solid phantom with a shorter T2 relaxation time does not have representative T2 relaxation time of muscle (35 ms at 1.5 T) [22]. While the phantoms investigated in this work are not representative for MRI properties of tissues of interest, their aimed application is QA studies with MR thermometry (MRT) validation [5]. Accuracy of MR-derived temperatures increases due to long T2 relaxation times, since temperature-to-noise ratio of MRT linearly depends on T2 relaxation times [24]. Therefore, water-based phantoms with long T2 relaxation times are suitable for validation of temperature or SAR patterns with MRT. 

### 3.2. Thermal Properties of the Three Phantoms Over Time

**Figure 2:**
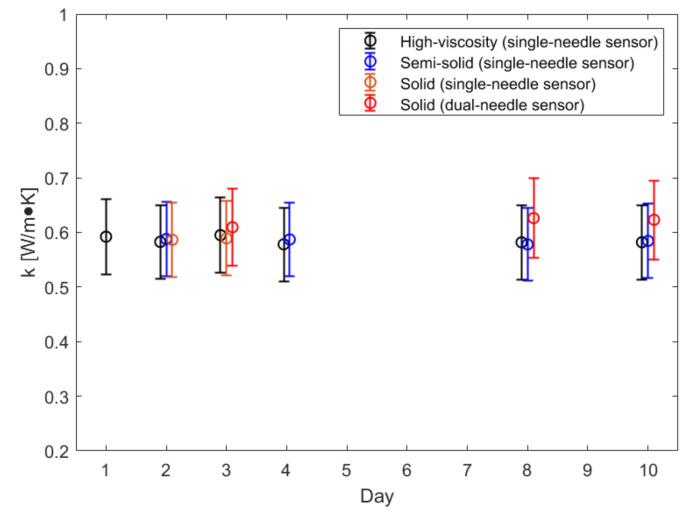
Thermal conductivity of the high-viscosity, semi-solid and solid phantom measured with the single-needle sensor and with the dual-needle sensor over time.

**Figure 3:**
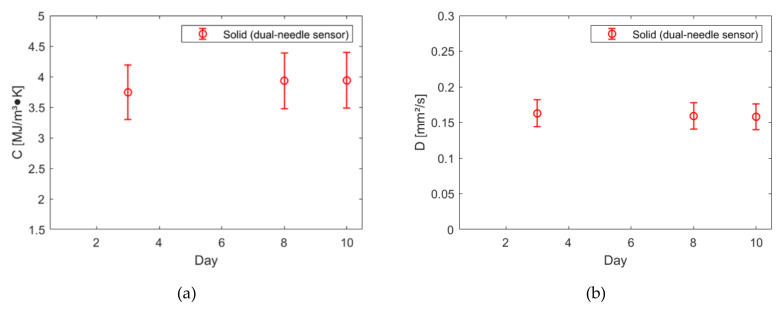
Volumetric heat capacity (**a**) and thermal diffusivity (**b**) of the solid phantom measured with the dual-needle sensor over time.

The measurements conducted on the phantoms over time (10 days) are illustrated in Figure 2 and Figure 3. In the figures, the average value measured each day is reported together with its associated uncertainty, calculated as in [16], i.e., accounting for the data variability and the device accuracy (10%). In Figure 2, the thermal conductivity of the three phantoms is shown. In Figure 3, the volumetric heat capacity and the thermal diffusivity of the solid phantom measured with the dual-needle sensor are shown.

For all the evaluated phantoms, the thermal conductivity values are in the range of those of body internal organs, such as liver (*k* = 0.48 ± 0.06 Wm^−1^K^−1^), kidney (*k* = 0.58 ± 0.07 Wm^−1^K^−1^) and muscle (*k* = 0.54 ± 0.06 Wm^−1^K^−1^); for the solid phantom, it is also possible to observe values comparable to the body tissue ones for volumetric heat capacity (liver: *C* = 3.27 ± 0.39 MJm^−3^K^−1^, kidney: *C* = 3.76 ± 0.44 MJm^−3^K^−1^, muscle: *C* = 3.61 ± 0.42 MJm^−3^K^−1^) and thermal diffusivity (liver, kidney and muscle: *D* = 0.15 ± 0.02 mm^2^s^−1^) [18]. The phantoms showed stability of their thermal properties over 10 days; the variability observed is minimal, lower than the uncertainty associated with the single measured values and negligible with respect to the accuracy of the device (10%). No difference in thermal conductivity has been observed for the three phantoms measured with the same sensor (single-needle), whereas the dual-needle sensor measured higher thermal conductivity values in the solid phantom with respect to the single-needle sensor (+0.03 Wm^−1^K^−1^). However, such difference (5%) is within the 10% accuracy of the device. 

### 3.3. Sensitivity Results

**Figure 4:**
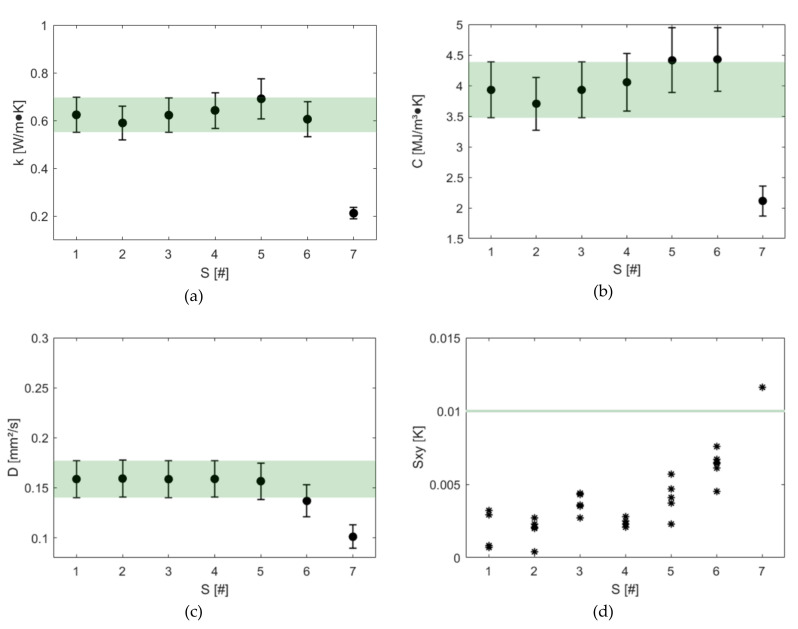
Sensitivity study on the dual-needle sensor: (**a**) thermal conductivity, (**b**) volumetric heat capacity, (**c**) thermal diffusivity and (**d**) regression error (Sxy).

The results of the sensitivity study conducted with the dual-needle sensor on the solid phantom are reported in Figure 4. Thermal conductivity, volumetric heat capacity and thermal diffusivity were measured on samples of decreasing size, as listed in Table 1.

The sensitivity study conducted on the solid phantom showed that the dual-needle sensor (SH-3) can correctly measure (within the 10% accuracy of the device indicated by the green bar in Figure 4) the thermal properties of a test sample smaller than the one specified by the manufacturer (i.e. 15 mm per side for a total of about 25 cm^3^). Reducing the radial dimension of the sample from 15 mm (S2 in Figure 4) to 8 mm per side (S4 in Figure 4), the device performances are secured; lack of accuracy can be observed between 8 mm and 4 mm (S6 in Figure 4), but the values measured remain within the uncertainty of the bulk data (S1 in Figure 4). When a minimum of 4 mm of material parallel to the sensor in all directions is not guaranteed (S7 in Figure 4), the sensor is not able to measure correctly. These results are also confirmed by Figure 4d, where we can observe how well the device’s least square model fits the data. It is worth to notice that reducing the dimensions of the sample, a longer time (up to 30 min) before performing the first measurement should be waited to allow the system to equilibrate. Last, the sample should always entirely cover the length of the sensor to ensure correct measurements, as inferred by the sensor measuring mechanism. Such conclusion has been experimentally confirmed: a 20 mm long solid phantom sample has been measured, and thermal conductivity and volumetric heat capacity values 30% lower (beyond the device accuracy) than in the bulk case (S1) have been obtained (*k* = 0.42 ± 0.05 Wm^−1^K^−1^, *C* = 2.66 ± 0.31 MJm^−3^K^−1^).

## 4. Conclusions

The three QA deep hyperthermia phantoms investigated in this study provide a comparable representation of the biological tissues they mimic (e.g., muscle) and are also similar to the reported values of internal organs such as liver or kidney, i.e., the high water content tissues: thermal properties of the phantoms are within the range of biological tissue, at room temperature. Thus, the phantoms are suitable for the evaluation of the performance of hyperthermia device, but they do not accurately simulate clinical scenarios, since their MR relaxation times are longer than the ones of biological tissues (e.g., muscle). For clinical applications that are sensitive to relaxation times such as MRT, dedicated MRI tissue representative phantoms are required.

The commercial dual-needle sensor investigated shows that to correctly measure samples as small as 2–5 cm^3^, at least 4 mm of material should be used in any direction parallel to the sensor.

## Figures and Tables

**Table 1:** Dual-needle sensor sensitivity study; measurements steps.

Name	Step	Dimension [mm] × [mm] × [mm]	Measurements
S1	bulk	>(30 × 36 × 30)	6
S2	15 mm	30 × 36 × 30	5
S3	10 mm	20 × 26 × 30	5
S4	8 mm	16 × 22 × 30	5
S5	6 mm	12 × 18 × 30	5
S6	4 mm	8 × 14 × 30	5
S7	2.5 mm	5 × 11 × 30	1

**Table 2:** Dielectric assessment of the phantoms at the frequencies of interest (70 MHz, 100 MHz and 434 MHz).

Phantom	Frequency	Dielectric Property	Measured Value	Reference Value
High-viscosity	70 MH	Relative permittivity	80.0	
		Conductivity [S/m]	0.45	
	100 MH	Relative permittivity	79.7	79.7 [5]
		Conductivity [S/m]	0.45	0.44 [5]
	434 MHz	Relative permittivity	78.4	
		Conductivity [S/m]	0.51	
Semi-solid	70 MH	Relative permittivity	77.7	75 [6]
		Conductivity [S/m]	0.60	0.55 [6]
	100 MH	Relative permittivity	77.8	
		Conductivity [S/m]	0.60	
	434 MHz	Relative permittivity	77.4	
		Conductivity [S/m]	0.66	
Solid	70 MH	Relative permittivity	77.0	
		Conductivity [S/m]	0.49	
	100 Mz	Relative permittivity	76.9	78.6 [7]
		Conductivity [S/m]	0.49	0.41 [7]
	434 MHz	Relative permittivity	76.6	
		Conductivity [S/m]	0.54	

**Table 3:** MR relaxation times of the phantoms at the 1.5 T.

Phantom	T1 (ms)	T2 (ms)
High-viscosity	2495 ± 92	870 ± 68
Semi-solid	2410 ± 247	742 ± 54
Solid	1917 ± 159	67 ± 1.8
